# Production of C, N Alternating 2D Materials Using Covalent Modification and Their Electroluminescence Performance

**DOI:** 10.1002/smsc.202000042

**Published:** 2021-01-15

**Authors:** Sunghee Park, Young-Hoon Kim, Sungwoo Kang, Donggyu Lim, Jinwoo Park, Dawoon Jang, Seungjoo Choi, Jeongho Kim, Seungwu Han, Tae-Woo Lee, Sungjin Park

**Affiliations:** ^1^ Department of Chemistry and Chemical Engineering Inha University 100 Inha-ro, Michuhol-gu Incheon 22212 Republic of Korea; ^2^ Department of Materials Science and Engineering Seoul National University 1 Gwanak-ro, Gwanak-gu Seoul 08826 Republic of Korea; ^3^ School of Chemical and Biological Engineering Seoul National University 1 Gwanak-ro, Gwanak-gu Seoul 08826 Republic of Korea; ^4^ Institute of Engineering Research Research Institute of Advanced Materials Nano Systems Institute (NSI) Seoul National University 1 Gwanak-ro, Gwanak-gu Seoul 08826 Republic of Korea

**Keywords:** carbon nitrides, density functional theory simulations, diazonium salts, light-emitting diodes, two-dimensional materials

## Abstract

Carbon nitrides (C_3_N_4_) show excellent properties in various optical and optoelectronic applications. However, their application to electroluminescence (EL) devices is limited by the lack of production methods of homogeneous dispersions in organic solvents, which are critically required for device fabrication. Herein, a strategy to generate stable dispersions of fluorescent, 2D C_3_N_4_ materials, and demonstrate light‐emitting diodes (LEDs) based on them is proposed. The treatment of urea‐driven C_3_N_4_ (UCN) with methoxy‐benzene diazonium salt (MD) produces dispersions in organic solvents. Experimental and theoretical studies suggest that MD treatment passivates the surface defects of the UCN. The resulting LED devices show bright green luminescence with an external quantum efficiency of 0.91%. This unprecedented result opens an era of C_3_N_4_ emitters as future promising light emitters in displays and solid‐state lighting.

## Graphical abstract

Stable dispersions of fluorescent, 2D C_3_N_4_ materials and light‐emitting diodes (LEDs) based on them are reported. The treatment of urea‐driven C_3_N_4_ with methoxy‐benzene diazonium salt produces dispersions in organic solvents and passivates the surface defects of C_3_N_4_ materials. LED devices show bright green light with an external quantum efficiency of 0.91%.

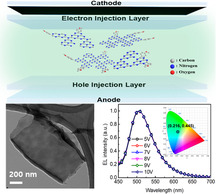

## 1 Introduction

Light‐emitting materials have been widely used in many applications such as displays, sensors, fluorescent probes, fashion textiles, and wearable devices. Especially, for display and lighting applications, the light‐emitting materials should have suitable bandgaps corresponding to visible light. Various semiconducting materials, such as inorganic nanoparticles,^[^ ^1^ ^]^ organic materials,^[^ ^2^, ^3^ ^]^ heavy‐metal‐based inorganic quantum dots (QDs),^[^ ^4^, ^5^ ^]^ and metal‐halide perovskites^[^ ^6^, ^7^, ^8^, ^9^, ^10^ ^]^ show excellent properties as a visible light‐emitting material. However, it is important to develop new materials that are environmental‐friendly, biocompatible, flexible, and cost effective for their possible applications in wearable and biocompatible devices.^[^ ^11^, ^12^, ^13^ ^]^

Recently, graphene QDs^[^ ^14^, ^15^ ^]^ and carbon nanodots^[^ ^16^ ^]^ have been studied as environmentally benign and biocompatible emitters to be used in both bioimaging and light‐emitting diodes (LEDs). However, these materials still have severe problems such as complex synthesis routes, low photoluminescence quantum efficiency (PLQE), excitation wavelength‐dependent PL spectra, injected current density‐dependent electroluminescence (EL) spectra, and low color‐purity of EL spectra (full width at half maximum [FWHM] > 150 nm).^[^ ^11^, ^14^, ^15^, ^16^ ^]^ In this regard, the metal‐free C, N alternating structures are interesting candidates due to their strong fluorescence and low cytotoxicity.^[^ ^12^, ^13^, ^17^, ^18^ ^]^

It is well known that 3D carbon nitrides (C_3_N_4_) can be easily produced by thermal condensation of small organic molecules.^[^ ^19^ ^]^ Optical characteristics of C_3_N_4_ originate from chemical structures of conjugated basic building units, typically *s*‐triazine and tris‐*s*‐triazine.^[^ ^20^, ^21^ ^]^ Their photophysical and porous properties lead excellent performance as photocatalysts, sensors, and adsorbents.^[^ ^22^, ^23^, ^24^ ^]^ However, the self‐quenching of luminescence in the 3D network and the poor solution processability induced by low dispersibility of the sp^2^ C–N network in organic solvents hamper them to be applied as light‐emitting materials in LEDs. The potential of C_3_N_4_‐based LEDs was demonstrated to achieve EL emission from C_3_N_4_ thin film which was directly grown on the indium tin oxide (ITO) surface.^[^ ^25^ ^]^ Although this work needs a high‐temperature (>450 °C) process and near‐infrared (NIR) EL emission came from discrete states (defect states or doping‐induced subenergy‐level states) which are generated from barbituric acid doping rather than the C_3_N_4_ emitter itself, this result suggests that C_3_N_4_ can be a strong candidate as a biocompatible emitter in practical applications of LEDs in displays and solid‐state lighting if one overcomes the high‐temperature process and achieves visible EL emission.

Recently, chemical modifications and insertion of small molecules into interlayer galleries of the 3D C_3_N_4_ network provided successful routes of exfoliation to produce 2D C_3_N_4_‐based nanodots in water.^[^ ^26^, ^27^, ^28^ ^]^ For example, the chemical oxidation of 3D C_3_N_4_ or attachment of a biocompatible and flexible group, polyethylene glycol (PEG), on C_3_N_4_ network produced 2D C_3_N_4_‐based materials. These 2D materials were dispersible and luminescent in aqueous media and showed good performances for optical cell imaging and low cytotoxicity.^[^ ^17^, ^18^, ^26^ ^]^ However, such aqueous suspensions are not suitable for the fabrication of LEDs because water can damage other layers such as the underlying hole‐injection interlayer (e.g., poly(3,4‐ethylenedioxythiophene): poly(styrenesulfonate) (PEDOT:PSS))^[^ ^27^ ^]^ and reduce luminescence efficiency and operating stability of LEDs.^[^ ^28^ ^]^ Some literatures reported the production of dispersions of covalently modified C_3_N_4_ materials or C_3_N_4_‐based hybrids in organic solvents.^[^ ^29^, ^30^, ^31^, ^32^, ^33^ ^]^ Although their luminescent properties were investigated intensively, the fabrication of efficient LEDs has not been reported yet.

**Figure 1:**
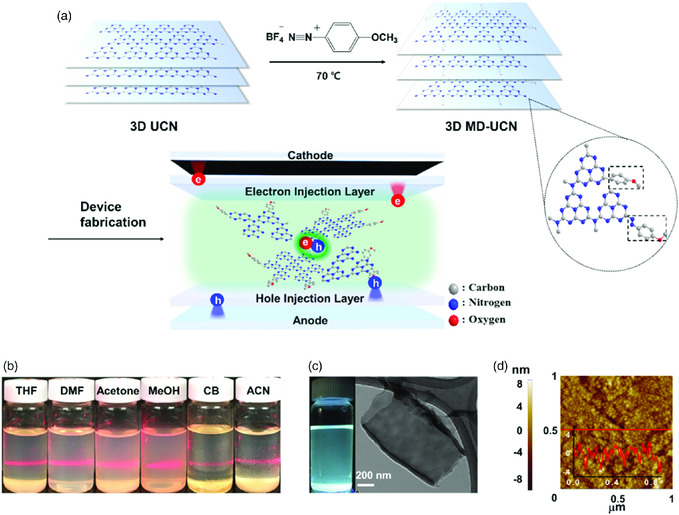
Overall concept of this work and characterizations of 2D C_3_N_4_‐based materials. a) A scheme for the production of MD–UCN and LED devices containing MD–UCN in the EML, b) colloidal dispersions (1 mg of MD–UCN per 15 mL of each solvent) of MD–UCN in organic solvents (THF, dimethylformamide [DMF], acetone, methanol [MeOH], chlorobenzene [CB], and acetonitrile [ACN]), c) a PL image of MD–UCN THF dispersion under a UV lamp (365 nm) and a TEM image of 2D MD–UCN, and d) an AFM image with a height profile of the MD–UCN film on the glass produced by spin‐coating of the THF dispersion.

In this work, we developed a new route to produce homogeneous colloidal dispersions of thin and fluorescent 2D C_3_N_4_ materials in organic solvents and successfully demonstrated efficient LEDs based on C_3_N_4_ materials (**Figure** **1**a). First, we chemically modified the surface of the C_3_N_4_ network, which was produced by thermal condensation of urea, with a methoxy‐benzene diazonium salt (MD). We, in accordance with density functional theory (DFT) calculations, show that the MD ligand significantly improves the dispersability and passivates the surface defects of 3D urea‐driven C_3_N_4_ (UCN), which show efficient excitation wavelength‐independent PL. With this simple but effective strategy, we achieved a current efficiency (CE) of 2.53 cd A^−1^, power efficiency (PE) of 1.59 lm W^−1^, and external quantum efficiency (EQE) of 0.91% in solution‐processed green‐emitting LEDs based on MD–UCN.

## 2 Results and Discussion

### 2.1 Production of 2D C_3_N_4_‐Based Materials and Their Characterizations

As shown in Figure 1a, the materials were produced by this sequence: 1) 3D UCN powder by thermal condensation of urea, 2) 3D MD–UCN powder by MD treatment of 3D UCN powder using a wet process, and 3) 2D MD–UCN suspensions by sonication of 3D MD–UCN powder. UCN powder was produced by thermal condensation of urea at 600 °C (Experimental Procedures, Supporting Information). The C_3_N_4_ network was modified by the chemical reaction of 3D UCN, which was produced by thermal condensation of urea, with a methoxy‐benzene diazonium salt. It is well known that functional groups of the diazonium salts easily modify sp^2^ carbons by C—C or diazonium couplings.^[^ ^34^, ^35^, ^36^ ^]^ The reactions can be done in water with/without the use of H_3_PO_2_.^[^ ^37^ ^]^ Thus, we tried aqueous reactions between 3D UCN and MD with and without H_3_PO_2_ at different temperatures (25, 40, and 70 °C). Among them, the reaction without H_3_PO_2_ at 70 °C produced the best result for improving THF dispersibility of the resulting materials. After purification using centrifugation with ethanol and vacuum drying, the final product (3D MD–UCN) was obtained as pale yellow powder.

Transparent, homogeneous, colloidal dispersions of 2D MD–UCN were produced by sonication of the 3D MD–UCN powder in various organic solvents (Figure 1b). We dispersed MD–UCN in tetrahydrofuran (THF) solvent because THF was reported to fabricate a uniform emitting layer (EML) without the aggregation of materials and dissolving other layers in LED devices.^[^ ^3^ ^]^ The stable dispersions showed the Tyndall effect, confirming the presence of light‐scattering particles. The THF dispersions were stable for at least 1 month (Figure S1, Supporting Information) and the maximum concentration was 3 mg per 7 mL. On the other hand, sonication of 3D UCN powder in THF produced heterogeneous mixtures containing floating particles rather than homogeneous dispersions (Figure S2, Supporting Information). The 2D MD–UCN was also well dispersed in various organic solvents such as methanol, acetonitrile, dimethylformamide, acetone, and chlorobenzene, as shown in Figure 1b, which indicates the versatility of MD–UCN in various wet processes. The THF dispersion of 2D MD–UCN showed fluorescence under a UV lamp (Figure 1c) and PLQE was measured to be 4.2%.

Transmission electron microscopy (TEM) samples were prepared by drying the droplets of 2D MD–UCN dispersions in THF on a lacey carbon TEM grid. As shown in TEM images (Figure 1c), overlapped thin layers with several hundred nm of length were found on the grid. On the other hand, TEM images of UCN displayed aggregated particles (Figure S3, Supporting Information), indicating improved dispersibility of 2D C_3_N_4_ materials by the MD treatment. An atomic force microscopy (AFM) image (Figure S4, Supporting Information) of the dried droplets of the dispersion on a mica disk showed the presence of thin layers with a thickness of 4–6 nm. The theoretical thickness of the monolayer of C_3_N_4_ is 0.33 nm and some experimental measurements reported that of ≈0.5 nm.^[^ ^38^, ^39^, ^40^ ^]^ However, chemical functionalization on the surface will increase the thickness. Although high‐purity graphene materials have ≈0.4 nm of thickness,^[^ ^41^, ^42^ ^]^ which is close to the theoretical value of 0.34 nm, chemically modified graphene‐based materials mostly showed a thickness of ≈1 nm.^[^ ^43^, ^44^, ^45^ ^]^ Consequently, the MD–UCN‐containing functional groups (see below for chemical characterizations) could have 4–6 layers of C_3_N_4_. Before applying the dispersions to LED fabrication, we tested the feasibility for the formation of a uniform film on a glass slide using spin coating. As shown in an AFM scan (Figure 1d), films with good surface uniformity (root mean square (RMS): 1.7 nm) were readily produced.

**Figure 2:**
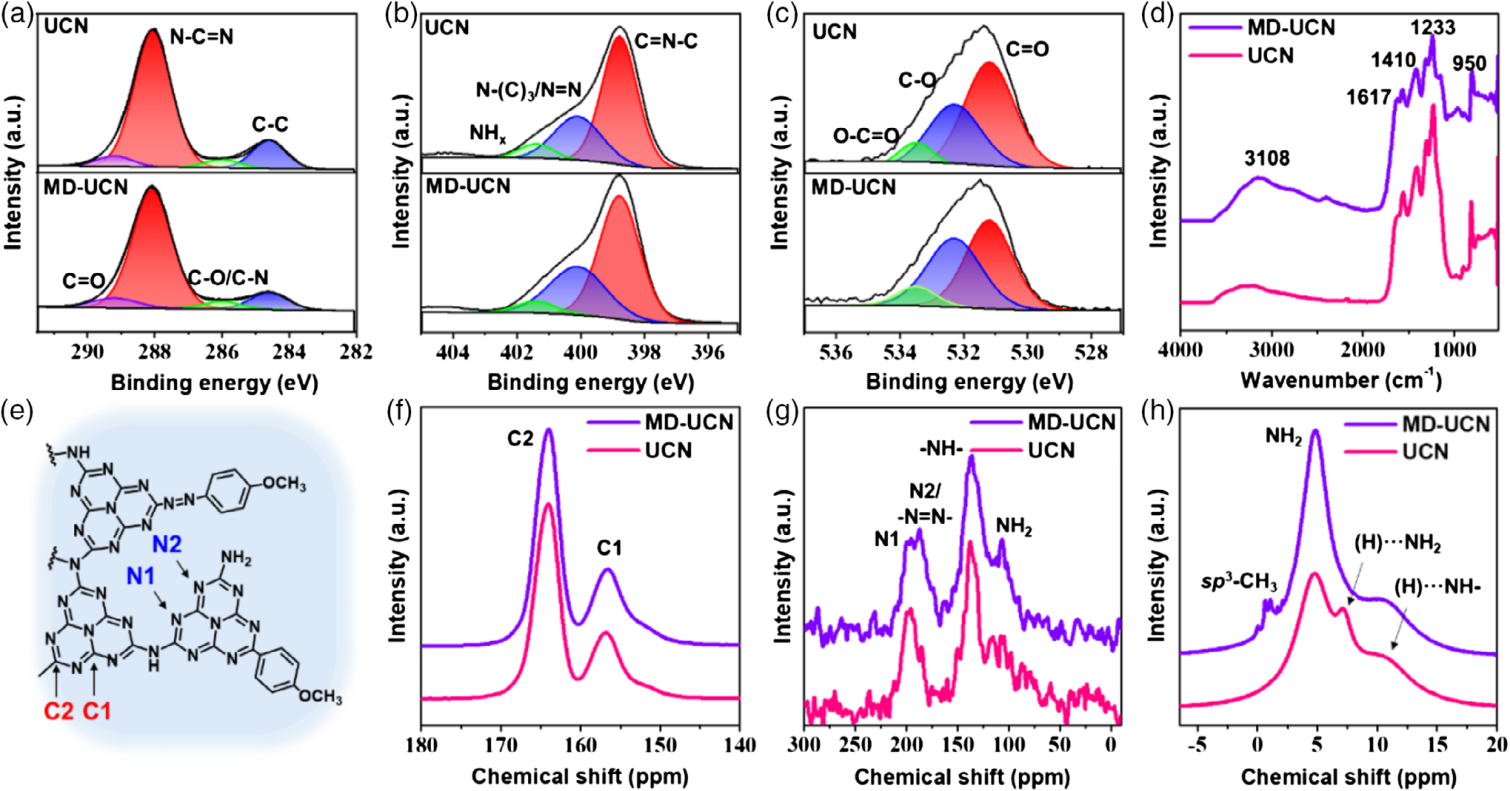
Deconvoluted XPS spectra of 3D UCN and 3D MD–UCN. a) C 1s, b) N 1s, c) O 1s, d) FTIR spectra of 3D UCN and 3D MD–UCN, e) a suggested chemical structure of 3D MD–UCN and SSNMR spectra of 3D ^13^C‐UCN and 3D ^13^C‐MD–UCN, f) ^13^C, g) ^15^ N, and h) ^1^ H.

We conducted chemical characterizations of MD–UCN and UCN (as a reference) to understand the improved dispersibility associated with the chemical structures. X‐ray diffraction (XRD) (Figure S5, Supporting Information), X‐ray photoelectron (XPS, **Figure** **2**a–c), and Fourier transform infrared (FTIR, Figure 2d) spectroscopy showed the formation of the C_3_N_4_ network in 3D UCN and 3D MD–UCN (see SI). UCN and MD–UCN powder samples showed broad XRD patterns typical for urea‐driven 3D C_3_N_4_‐based materials with a broad peak at around 27°, corresponding to an interplanar distance between C_3_N_4_ layers (Figure S5, Supporting Information).^[^ ^46^ ^]^ The XPS C 1s spectrum showed a large peak at 288.2 eV, which corresponds to the N=C—N moieties in the triazine building units (Figure 2a).^[^ ^47^ ^]^ The deconvoluted N 1s spectrum of UCN showed peaks at 398.8, 400.1, and 401.4 eV, corresponding to the C=N—C, —N=N—/N—(C)_3_, and —NH_*x*_, respectively (Figure 2b).^[^ ^46^ ^]^ The FTIR spectrum of the 3D UCN showed peaks at 1400–1650 cm^−1^, corresponding to heptazine‐derived repeating units, and at 1321 and 1250 cm^−1^, corresponding to completely condensed C—N and partially condensed C—NH moieties, respectively (Figure 2d).^[^ ^38^ ^]^ All these characterizations indicate the generation of the C_3_N_4_ network in the UCN.

After the reaction between MD and UCN powder, the FTIR spectrum of the 3D MD–UCN showed increased intensity of the peaks at around 950, 1617, and 3108 cm^−1^, corresponding to =C—H bending, C=C/N=N groups, and =C—H stretching, respectively (Figure 2d).^[^ ^48^ ^]^ It suggests the attachment of phenyl groups to the UCN network. The intensity of the peaks at 1410 and 2777 cm^−1^, corresponding to C—O/C—N groups and aliphatic —C—H stretching, also increased, indicating the presence of —OCH_3_ groups.^[^ ^38^, ^48^ ^]^ The XPS measurements of 3D MD–UCN found increased amount of O atoms after the MD modification (Table S1, Supporting Information). Deconvoluted C 1s and O 1s spectra showed that the intensity of the C—O peak increased and deconvoluted N 1s spectra showed the increased intensity of —N—(C)_3_/—N=N— peak (Figure 2a,c and Table S2, Supporting Information). The intensity of the XPS N 1s peak, corresponding to —N—(C)_3_/—N=N—, was enhanced after MD modification (Figure 2b, Table S3, Supporting Information). These results indicated that MD moieties were successfully attached to the surface of UCN.

Because nuclear magnetic resonance (NMR) spectroscopy can detect ^13^C isotope, the ^13^C‐labeled UCN (^13^C‐UCN) powder was prepared by the thermal condensation of ^13^C‐urea and ^13^C‐MD–UCN powder, which was obtained from the reaction between MD and ^13^C‐UCN. The ^13^C‐solid‐state NMR (SSNMR) spectra of the 3D ^13^C‐UCN and 3D ^13^C‐MD–UCN showed two peaks for C1 and C2 moieties, which correspond to internal and edge C atoms, respectively (Figure 2e,f).^[^ ^49^ ^]^ After MD modification, the C2/C1 ratio increased from 2.27 (^13^C‐UCN) to 2.80 (^13^C‐MD–UCN), suggesting the formation of more edge C atoms and more 2D C_3_N_4_, which was exfoliated from 3D C_3_N_4_. The ^1^H‐ and ^15^N‐SSNMR measurements showed obvious changes in the chemical structures of C_3_N_4_ after MD modification. The ^15^ N‐SSNMR spectra of ^13^C‐UCN and ^13^C‐MD–UCN showed the presence of N1, N2, –NH–, and –NH_2_ (Figure 2g).^[^ ^49^ ^]^ After MD modification, a peak for –NH_2_ and a shoulder peak for N2 increased; these can be explained by the formation of edge NH_2_ groups. In addition, the shoulder peak can be assigned to the formation of —N=N— bridges, as discussed from the XPS data (see Supporting Information).^[^ ^50^ ^]^ The ^1^ H‐SSNMR spectrum showed new peaks around 0–1 ppm, which correspond to the sp^3^C–H moieties, after MD modification (Figure 2h).^[^ ^51^ ^]^ The ^1^ H‐SSNMR spectrum showed an increased peak at ≈5 ppm, corresponding to —NH_2_ without hydrogen bonding, but a decreased peak at ≈7 ppm, corresponding to —NH_2_ with hydrogen bonding;^[^ ^49^ ^]^ these suggest that lots of H‐bonding in the 3D C_3_N_4_ structure were broken and methoxy groups in MD were chemically attached to the broken H‐bonding in the 2D UCN surface after MD modification through covalent bonding (will be described in the simulation section). This MD modification passivates the broken H‐bonding, which acts as defect sites and results in decent luminescence efficiency of 4.2% for MD–UCN. The NMR and XPS data indicated the presence of —N=N— bridges in the MD–UCN sample. The methoxy‐phenyl groups were introduced by the C—C coupling reaction through carbocations and/or by the azo‐coupling reaction (Figure S6, Supporting Information),^[^ ^52^ ^]^ which produces a —N=N— bridge between C_3_N_4_ and functional groups. All these changes indicate that the CH_3_–O–phenyl groups were attached on the edges of C_3_N_4_ network by covalent bonds. These C_3_N_4_ networks decorated with the functionalities showed enhanced dispersibility, resulting in the production of stable homogeneous colloidal dispersions.

**Figure 3:**
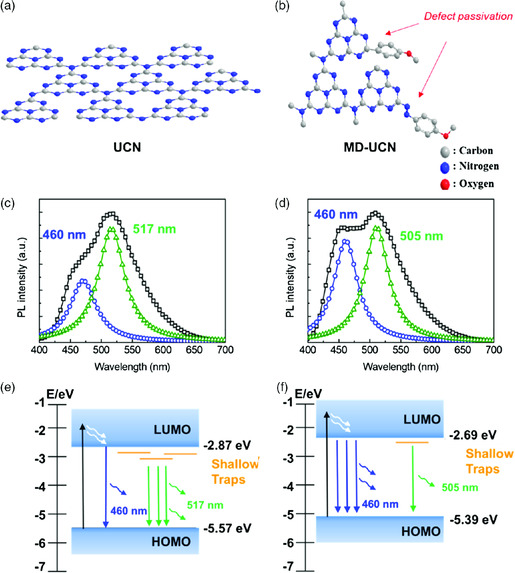
Chemical structures of a) UCN and b) MD–UCN. Photophysical properties of 2D UCN and 2D MD–UCN. Deconvoluted PL spectra of c) 2D UCN and d) 2D MD–UCN. Schematic illustrations of the energy structure and emission mechanisms of e) UCN and f) MD–UCN.

To understand the effects of MD treatment on the photophysical properties of UCN emitters, we measured ultraviolet photoelectron spectroscopy (UPS), UV–Vis absorption, PL, and PL excitation (PLE) spectroscopy of 2D UCN and 2D MD–UCN emitters (Figure S7a‐d, Supporting Information). The UV–Vis absorption and PLE spectra of 2D MD–UCN dispersions were similar to those of UCN. However, the PL spectrum of MD–UCN dispersions was blue‐shifted and showed an enhanced peak at ≈460 nm relative to that of UCN, indicating that MD passivates the defects in UCN emitters rather than electronic coupling (orbital hybridization) with UCN emitters (**Figure** **3**a–d).^[^ ^53^ ^]^ From the earlier results, a schematic illustration of the electronic structures of UCN‐based emitters can be proposed to explain their emission mechanisms (Figure 3e,f, and S7e,f, Supporting Information). Here, energy levels of emitters were determined by UPS and optical absorption data (Figure S8, Supporting Information). The 2D MD–UCN also showed a higher photostability than 2D UCN (Figure S9, Supporting Information) and excitation wavelength‐independent PL emission (Figure S7a,c, Supporting Information). These phenomena are important to maintain stable luminescence in solid‐state lighting and display applications, which are different from graphene QDs and carbon nanodots of which PL wavelength changes depending on the excitation wavelength.^[^ ^11^, ^14^, ^15^, ^16^ ^]^

**Figure 4:**
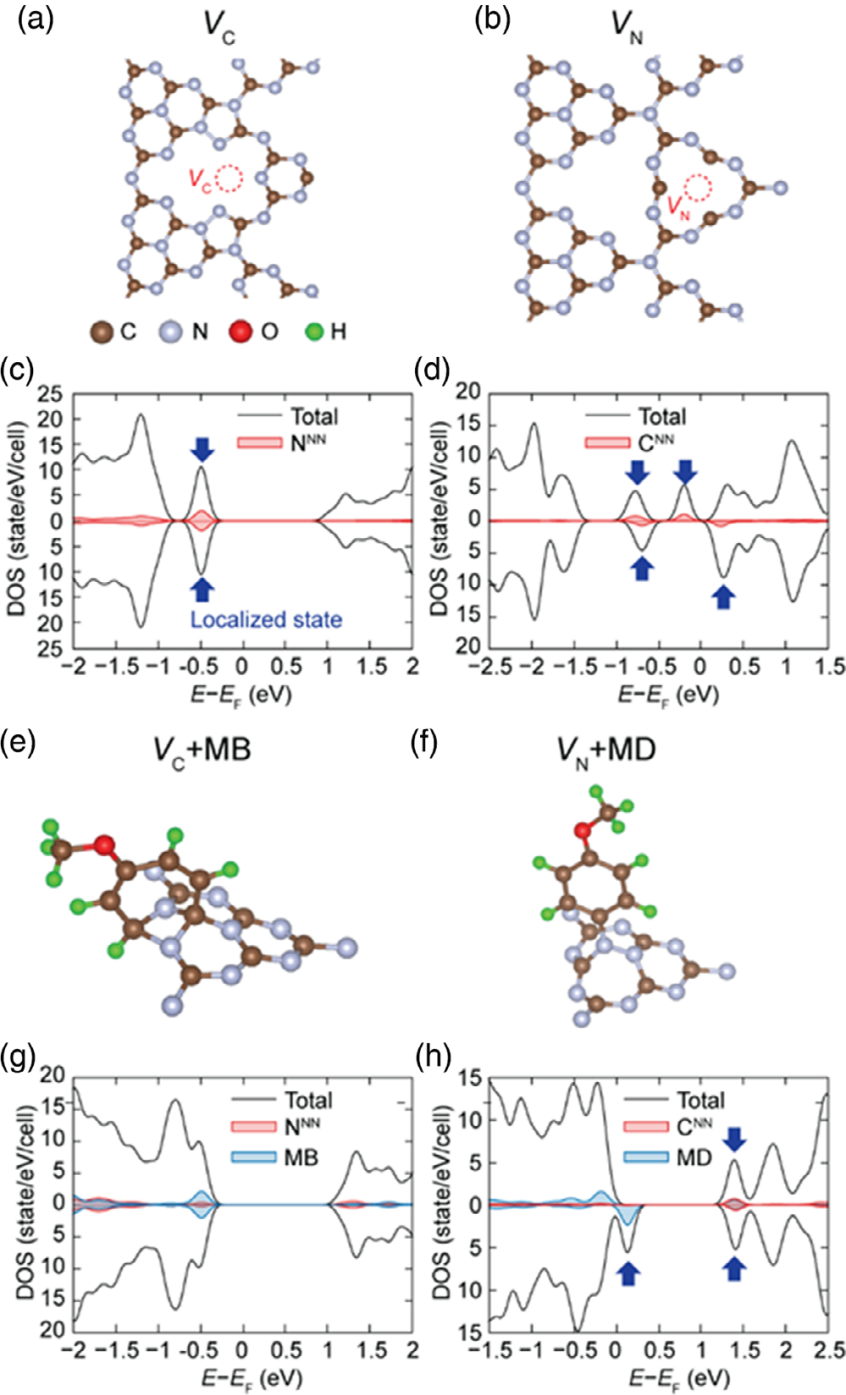
DFT and DOS calculations of UCN and MD–UCN vacancy. a,b) Atomic structures and c,d) spin‐resolved DOS of *V*_C_ and *V*_N_, respectively. Vacancy sites are indicated with a dotted circle. The blue arrows in DOS indicate the localized states (see Figure S10, Supporting Information, for partial charges). e,f) Atomic structures and g,h) spin‐resolved DOS of *V*_C_ + MB and *V*_N_ + MD, respectively. Partial DOS of nearest‐neighbor N (N^NN^), C^NN^, MB and MD are plotted (c, d, g, and h) with total DOS. All energies in (c, d, g, and h) are offset by Fermi levels (*E*_F_).

We carried out DFT calculations to further understand the role of MD on defect passivation. As both C and N vacancies (*V*_C_ and *V*_N_, respectively) were found in C_3_N_4_‐based materials,^[^ ^54^, ^55^, ^56^ ^]^ we considered both types of vacancies. Among the possible vacancy configurations, we chose the energetically most favorable ones, as shown in **Figure** **4**a,b, respectively. The corresponding density of states (DOS) are shown in Figure 4c,d. The localized midgap states associated with defects were identified for both vacancies (see blue arrows). These states are localized around the vacancies which may act as trap states (see Figure S10, Supporting Information), which are consistent with the schematic diagram in Figure 3e.

To investigate the interaction between MD and vacancies, we calculated free energies of various structures of vacancies with chemical adsorption of the MD ion, and those with chemical adsorption of methoxy‐benzenium (MB) ion (see Figure S6, Supporting Information), and chose energetically favorable configurations (see Supporting Information for details). Figure 4e,f shows the most stable structures for *V*_C_, and *V*_N_, respectively, which show that MB and MD ions are covalently attached on *V*_C_ and *V*_N_, respectively. The DOS of MB‐adsorbed *V*_C_ (*V*_C_ + MB) and MD‐adsorbed *V*_N_ (*V*_N_ + MD) are shown in Figure 4g,h, respectively. The adsorption of MB by the covalent bond completely reduced the defect states of *V*_C_, and therefore, the highest occupied molecular orbital (HOMO) becomes delocalized, as shown in Figure S10d, Supporting Information. This is consistent with the improvement of optical properties of 2D MD–UCN compared with 2D UCN as shown in Figure 3f. On the other hand, the defect states of *V*_N_ were not perfectly passivated by adsorption of MD, as shown in Figure 4h (see Figure S10e, Supporting Information). This might explain the remained defect‐related PL peak of 2D MD–UCN (Figure 3d). We expect that the combination of MD with other molecules passivating *V*_N_ can further improve the performance of C_3_N_4_, which might be the subject of the future study. These defect passivation effects of MD on UCN can be confirmed by the increase in PL lifetime in 2D MD–UCN (Figure S11, Supporting Information).

### 2.2 Fabrication of LEDs and EL Performances

**Figure 5:**
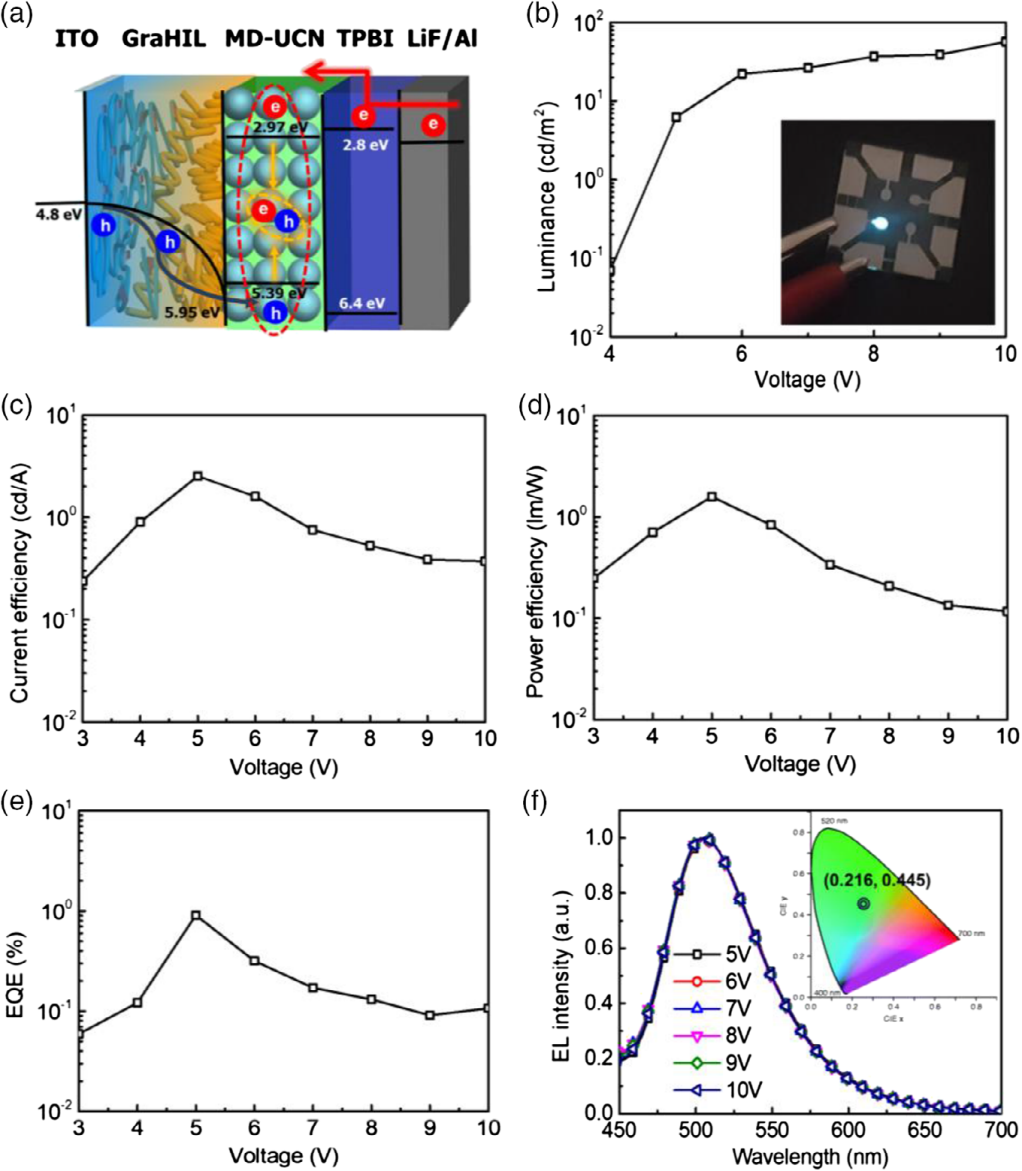
EL performances of the LEDs. a) A band energy diagram of the device, b) luminance versus voltage characteristic (inset: a photo of an LED device), c) CE versus voltage characteristic, d) PE versus voltage characteristic, e) EQE versus voltage characteristic, and f) EL spectra with different applied biases and the corresponding CIE diagram of MD–UCN‐based LEDs (inset).

Using the 2D MD–UCN film as an EML, we successfully fabricated LEDs that include a self‐organized polymeric gradient hole injection layer (GraHIL)^[^ ^3^, ^57^ ^]^ and 1,3,5‐tris(*N*‐phenylbenzimidizol‐2‐yl)benzene (TPBI) as an electron transport layer (ETL) (**Figure** **5**a). The GraHIL consists of PEDOT:PSS and perfluorinated polymeric acid, tetra‐fluoroethylene‐perfluoro‐3,6‐dioxa‐4‐methyl‐7‐octene‐sulfonic acid copolymer (PFI). A relatively deeper valence band maximum (*E*_v_) of MD–UCN (5.39 eV), compared with the work function (WF) of conventional PEDOT:PSS (4.80 eV), can induce a hole injection barrier (0.59 eV). This can limit the hole injection and charge balance in the MD–UCN EML and thus, reduce the EL efficiency in LEDs. These problems were solved by the use of GraHIL conducting polymer compositions. During the fabrication of GraHIL, the concentration of PFI gradually increased from the bottom surface to the top surface in GraHIL because PFI had a lower surface energy (20 mN m^−2^) than PEDOT:PSS (38 mN m^−2^).^[^ ^3^, ^57^ ^]^ The self‐organization of PFI induced a gradual increase of the ionization energy from the bottom (4.80 eV) to the top (5.95 eV) because PFI has deep ionization energy (6.10 eV). This gradient ionization energy can facilitate hole injection from the ITO electrode to the MD–UCN EML (*E*_v_: 5.39 eV) by reducing the hole injection barrier (Figure 5a). Surface‐enriched PFI can also prevent exciton quenching, which easily occurs at the interface between PEDOT:PSS and MD–UCN EML and consequently improves the device efficiency and luminance of MD–UCN‐based LEDs.^[^ ^3^, ^57^ ^]^ TPBI has a deep HOMO level (6.40 eV) and a shallow lowest unoccupied molecular orbital (LUMO) level (2.80 eV), which facilitate electron injection into the MD–UCN EML (conduction band minimum [*E*_c_]: 2.97 eV) and confine the exciton in the EML. The average thickness of the MD–UCN EML measured by ellipsometry measurements was 40 nm.

As we applied an electrical bias on LEDs, electrons and holes are injected through LiF/Al cathodes and ITO anodes, respectively. These electrons and holes are transported into the MD–UCN EML via TPBI and GraHIL, respectively, and then recombine to emit visible light, which has a similar energy with the bandgap of MD–UCN. Generated photons were emitted through the transparent ITO electrode and showed bright EL emission (Inset of Figure 5b). MD–UCN‐based LEDs showed a CE of 2.53 cd A^−1^, PE of 1.59 lm W^−1^, maximum luminance of 56.75 cd m^−2^, and EQE of 0.91% (Figure 5b–f, and S12, Supporting Information). The measured EQE is well matched with a theoretical calculation value (EQE (1.05%) = 4.2% (PLQE of emitter) × 0.25 (outcoupling factor) × 1 (charge balance factor) × 1 (probability of production of emissive species)). To our best knowledge, this is the first efficient LED based on thin C_3_N_4_ emitters to date and our CE is 28 times and 180 times improvement over the previously reported C_3_N_4_‐based LEDs.^[^ ^58^, ^59^ ^]^ The EL spectrum of the LEDs showed a single green emission peak at 506 nm (Figure 5f), which are different from the PL spectrum showing a shoulder peak at 460 nm. This difference could be attributed to the energy transfer of excitons to lower energy sites under an electrical field^[^ ^60^, ^61^ ^]^ and thus, photons with a relatively lower energy were emitted in C_3_N_4_ emitters. It can be supported by the longer PL lifetime (0.55 ns) measured at 502 nm than that at 450 nm (0.14 ns) (Figure S13, Supporting Information). The FWHM of the EL peak was 76 nm, which is significantly smaller than that of other biocompatible emitters such as graphene QDs (>150 nm)^[^ ^14^, ^15^ ^]^ and carbon nanodots (>100 nm);^[^ ^11^, ^16^ ^]^ it indicates that the MD–UCN emitters can emit a higher color‐purity light and have more possibilities to be used in vivid natural color displays compared with graphene QDs and carbon nanodots.

The Commission Internationale de l’Éclairage (CIE) coordinates at (0.216, 0.445) and EL spectrum do not change according to the applied bias (Figure 5f, inset), which is contrary to the graphene QDs and carbon nanodots;^[^ ^11^, ^14^, ^15^, ^16^ ^]^ these mean that GraHIL and TPBI efficiently transport and confine charge carriers inside the MD–UCN EML and the EL emission of our LEDs generated from pure MD–UCN. These device efficiencies are comparable with those of state‐of‐the‐art biocompatible emitters (EQE of 2.67% for graphene QDs^[^ ^14^, ^15^, ^62^ ^]^ and 0.6% for carbon nanodots^[^ ^16^ ^]^). The conventional environmentally benign light‐emitters (graphene QDs, carbon nanodots) have drawbacks in lighting technologies: 1) excitation wavelength‐dependent PL wavelength, 2) undesired change of EL wavelength depending on the injected current density, and 3) low color purity limiting their applications in vivid display technology.^[^ ^11^ ^]^ However, our MD–UCN‐based devices showed promising results to solve such problems as discussed earlier.

Because the preparation of MD–UCN materials and fabrication of the devices are not optimized, the PLQE of the emitters and EQE of their LEDs are still far below industrially viable requirements. However, the long‐term history tells us that dramatic improvements of EQEs have been made in organic emitters from 2.5%^[^ ^2^ ^]^ to 35.6%,^[^ ^63^ ^]^ in inorganic QDs from 0.52%^[^ ^64^ ^]^ to 22.9%,^[^ ^65^ ^]^ in metal‐halide perovskites from 0.1% to 21.6%,^[^ ^6^, ^9^, ^10^, ^66^ ^]^ and in graphene QDs from 0.1%^[^ ^14^ ^]^ to 1.28%.^[^ ^15^ ^]^ Thus, we believe that this result would open a new era of light emitters and EQE will be greatly improved by further optimizing synthetic routes, chemical defect passivation, organic host‐C_3_N_4_ guest systems for efficient energy transfer, and device fabrication processes. Furthermore, it is important to note that our development uses low‐temperature wet processes and shows bias‐independent bright and visible EL emission in LEDs, which solve the limitations of previously reported C_3_N_4_‐based LEDs, such as high synthesis temperature, applied bias‐dependent EL emission with low luminescence efficiency, and NIR emission.^[^ ^25^ ^]^

## 3 Conclusion

In this work, homogeneous colloidal dispersions of thin, metal‐free, and fluorescent C_3_N_4_ materials (MD–UCN) were produced in organic solvents, and efficient LEDs based on them were fabricated using a wet process. To improve dispersibility and passivate the defects, the C_3_N_4_ network was modified with methoxy‐phenyl groups by the reaction between 3D UCN powder and MD. The chemical characterization of 3D MD–UCN found that the C_3_N_4_ network was decorated by methoxy‐phenyl groups through covalent bonds. Using the fluorescent dispersions including 2D MD–UCN, we fabricated LEDs that showed promising EL efficiencies with a CE of 2.53 cd A^−1^, PE of 1.59 lm W^−1^, and EQE of 0.91%. To our best knowledge, this is the first reported EL efficiencies in thin C_3_N_4_ emitters to date. Therefore, we believe that our report will open a new era of C_3_N_4_ emitters as promising light‐emitters in displays and solid‐state lighting. Furthermore, because of excellent biocompatibility and low cytotoxicity of the C_3_N_4_ network, the C_3_N_4_ emitter will be also highly useful for biocompatible electronic devices.

## 4 Experimental Section

4.1

4.1.1

##### Preparation of 3D UCN

An alumina crucible filled with urea powder (10 g, 99%, Sigma Aldrich) was placed in the middle of a quartz tube in a furnace (TFP‐80‐3, Dongseo Science co., Ltd., Korea). The temperature of the furnace was elevated to 600 °C with a heating rate of 3 °C min^−1^, under N_2_ flow, and held at 600 °C for 2 h. Then the quartz tube was cooled down to room temperature, affording pale yellow powder (UCN).

##### Preparation of 3D MD–UCN

The as‐prepared UCN powder (35 mg) was added into a round‐bottom flask filled with 30 mL of distilled water; then, the mixture was sonicated for 15 min. The flask was soaked into a water bath heated at 70 °C and 4‐methoxy‐benzene diazonium tetrafluoroborate (500 mg, 98%, Sigma‐Aldrich) was added to the flask. The reaction mixture was stirred with a magnetic bar for 2 h. Then, distilled water (50 mL) was added to lower the reaction temperature. Resulting suspensions were centrifuged for 5 min at 10 000 rpm with a centrifuge (Supra 22 K, Hanil science Inc., Korea). Supernatant was decanted, then the wet precipitate was washed with ethyl alcohol (anhydrous, 99.9%, Daejung) using a centrifuge three times. The final precipitate was dried under vacuum at room temperature for 12 h, affording the pale yellow powder (MD–UCN, 25 mg).

##### Preparation of ^13^C Isotope‐Labeled 3D UCN and 3D MD–UCN and NMR Analyses

The ^13^C‐labeled 3D UCN (^13^C‐UCN, 50 mg) was prepared from urea‐^13^C (99.0 at% ^13^C, Sigma‐Aldrich) using the same procedure for UCN. The ^13^C‐labeled 3D MD–UCN (^13^C‐MD–UCN, 40 mg) was produced by the reaction between ^13^C‐UCN and MD as described earlier. All of the SSNMR experiments were conducted at a static magnetic field of 9.4 T with a Bruker Avance III HD NMR spectrometer with a Bruker 4 mm HX CPMAS probe (400.25 MHz) at a zirconia rotor. All of the ^13^C chemical shifts were referenced to the trimethylsilane (TMS) peak at 0 ppm, and the ^15^ N chemical shifts were referenced to NH_4_Cl at 39.1 ppm with a scale of liquid NH_3_. The ^15^ N NMR spectra were obtained with these acquisition parameters (number of scans: 23 223 times, spectral width in hertz: 32 467 Hz, acquisition time: 0.0149 s, receiver gain: 71.34, dwell time: 15.4 μ, and prescan delay: 10.81 μc). The ^13^C NMR spectra were obtained with these acquisition parameters (number of scans: 128 times, spectral width in hertz: 39 062 Hz, acquisition time: 0.0119 sec, receiver gain: 87.59, dwell time: 12.80 μs, and prescan delay: 8.0 μc). The temperature, Larmor frequency, and a spinning rate were set to 300 K, 100.66 MHz, and 10 KHz, respectively.

##### DFT Calculations

DFT calculations were carried out using the Vienna Ab initio Simulation Package (VASP) with projector augmented wave (PAW).^[^ ^67^, ^68^ ^]^ The generalized gradient approximation (GGA) was used for the exchange‐correlation functional.^[^ ^69^ ^]^ The energy cutoff for the plane‐wave basis was set to 450 eV after being tested. The simulations were conducted in a 12.35 Å × 14.27 Å supercell, consisting of eight C_3_N_4_ units. About 20 Å of the vacuum region was inserted to avoid spurious interactions among periodic images. The 1 × 1 × 1 (Γ‐point) and 3 × 3 × 1 k‐point meshes were used for structural relaxation and DOS calculations, respectively. The atomic structures of each simulation cell were optimized until forces of all the atoms were reduced within 0.03 eV Å^−1^.

To compare the relative energies (*E*_rel_) between MD and MB adsorbed vacancies, the free energies are calculated as follows

(1) $$E_{\text{rel}}=E(V_{\text{C,N}}+MD)-E(V_{\text{C,N}}+MB)-2\mu (N)$$

where *μ*(N) is the chemical potential of N. As the reference state, we choose N_2_(g) and graphite for N and C, respectively. As the charges, geometries, and dielectric constants are similar for *V*_C,N_ + MD and *V*_C,N_ + MB, we did not include any charge corrections. *E*_rel_ of *V*_N_ was −0.86 and 0.46 eV in N‐rich and C‐rich conditions, respectively, and *E*_rel_ of *V*_C_ was 2.54 eV and 3.86 eV in N‐rich and C‐rich condition, respectively. Therefore, we chose the adsorption of MD and MB for *V*_C_ and *V*_N_, respectively, as discussed in the main text.

##### Fabrication and Characterization of MD–UCN‐Based LEDs

ITO (180 nm)‐patterned glasses were cleaned by sonication in acetone and 2‐isopropanol and boiled in 2‐isopopanol for 15 min, respectively. Dried ITO‐patterned glasses were treated with UV–ozone to remove the residual dusts and make the surface hydrophilic. Then, GraHIL, which contained PEDOT:PSS and PFI with 1:1 weight ratio, was spin coated with 4500 rpm to give a thickness of 40 nm and baked at 150 °C for 30 min to remove the residual solvent. After finishing the baking process, substrates were moved to the N_2_‐filled glove box, and then, 2D MD–UCN suspension, which was diluted in THF, was spin coated with 1000 rpm for 60 s to give thickness of 40 nm, which is similar with recent LEDs based on colloidal nanocrystals.^[^ ^9^, ^10^ ^]^ After being annealed at 90 °C for 20 min, TPBI (50 nm), LiF (1 nm), and Al (100 nm) were deposited sequentially in a high‐vacuum chamber (<10^−7^ Torr). The current–voltage–luminance characteristics of MD–UCN‐based LEDs were measured using a Keithley 236 source measurement unit and a Minolta CS2000 spectroradiometer.

## Conflict of Interest

The authors declare no conflict of interest.

## Supporting information

Supplementary Material
