# Supplementary material for: Production of C, N Alternating 2D Materials Using Covalent Modification and Their Electroluminescence Performance
Source: Small Sci. 2021 Jan 15;1(2):2000042. doi: 10.1002/smsc.202000042 (PMC11935893; doi:10.1002/smsc.202000042)
Supplement: Supplementary file 1 — Supplementary Material [file SMSC-1-2000042-s001.docx]

Supporting Information

**Production of C, N Alternating Two-Dimensional Materials using Covalent Modification and Their Electroluminescence Performance**

*Sunghee Park, Young-Hoon Kim, Sungwoo Kang, Donggyu Lim, Jinwoo Park, Dawoon Jang, Seungjoo Choi, Jeongho Kim, Seungwu Han, Tae-Woo Lee,* and Sungjin Park**

**Instruments and measurements**

X-ray photoelectron spectroscopy (XPS) measurements were performed using a high performance X-ray photoelectron spectrometer (K-ALPHA HP-XPS, Thermo Fisher Scientific, U.K.) equipped with an monochromated X-ray source 400-µm 12 kV, 72W spectrometer. The Fourier transform infrared (FT-IR) spectra were obtained with KBr pellets containing powder samples using an FT-IR Vacuum Spectrometer (Bruker VERTEX 80V, Bruker, Germany). Transmission electron microscopy (TEM) measurements were performed with JEM2100F (JEOL, Japan) and JEM-ARM200F (JEOL, Japan) at 200 kV with samples on a carbon/copper grid (HC200-Cu, EMS, USA). UV-visible absorption of the samples was measured using a UV-visible spectrophotometer UV-2600 (Shimadzu, Japan). Atomic force microscopy (AFM) samples were prepared on mica disks (quality grade V-1 highest, 9.9 mm diameter TED PELLA, USA) and images were obtained using a Multimode 8 (Bruker, Germany). Ultraviolet photoelectron spectroscopy (UPS) data were measured with an Ultraviolet photoelectron spectrometer (AXIS-NOVA, KRATOS Inc.) using a monochromatic Al-Ka photon source with an analyzer pass energy set to 5 eV for He I. X-ray diffraction (XRD) patterns were obtained with a Multi-Purpose X-ray Diffractometer (X'Pert Powder Diffractometor-PANalytical). Photoluminescence (PL) and PL excitation (PLE) spectra were measured using a JASCO FP8500 spectrofluorometer. PL quantum efficiency (PLQE) was measured using a JASCO FP8500 spectrofluorometer with a 100-nm integrating sphere (ILF-835) and calculated in Jasco SpectraManager II Software. The excitation wavelength for measuring PLQEs was 370 nm which induced maximum PL intensity. PL lifetimes of samples were detected by micro-channel-plate photomultiplier tube (MCP-PMT) (R3809U-50, Hamamatsu) with time-correlated single photon counting (TCSPC) module (PicoHarp, PicoQuant). A picosecond-pulse laser head (LDH-P-C-405B, PicoQuant) with 405-nm excitation wavelength was used as an excitation source. The thickness of MD-UCN films was measured by using spectroscopic ellipsometer (m2000, Ja Woollam, Japan). To increase the reliability, data were measured at different angles (65°, 70°, and 75°) and fitted with b-spline model.

**Results and discussion**

**Preparation of materials**

To generate homogeneous colloidal dispersions of C_3_N_4_-based materials in THF, various 3D C_3_N_4_ samples were prepared by thermal condensation of common precursors (melamine, dicyandiamide, and urea). However, even lengthy sonication of the 3D C_3_N_4_ samples using an ultrasound bath was not successful to produce stable homogeneous dispersions in THF. We also tested the oxidized^[1]^ or PEGylated C_3_N_4_ samples,^[2]^ which were successfully dispersed in water, however, no stable dispersions in THF were generated.

The C_3_N_4_ network was modified by the chemical reaction of 3D urea-driven C_3_N_4_ (UCN), which was produced by thermal condensation of urea, with a methoxy-benzene diazonium salt (MD) (Figure S1). It is well-known that functional groups of the diazonium salts easily modify *sp*^2^ carbons by C–C or diazonium couplings.^[3-5]^ The reactions can be done in water with/without the use of H_3_PO_2_.^[6]^ Thus, we tried aqueous reactions between 3D UCN and MD with and without H_3_PO_2_ at different temperatures (25, 40, and 70 °C). Among them, the reaction without H_3_PO_2_ at 70 °C produced the best result for improving THF-dispersibility of the resulting materials. After purification using centrifugation with ethanol and vacuum-drying, the final product (MD-treated UCN, MD-UCN) was obtained as pale yellow powder.

**Chemical characterizations**

3D UCN and 3D MD-UCN samples showed broad X-ray diffraction (XRD) patterns typical for urea-driven 3D C_3_N_4_-based materials with a broad peak around 27^o^, corresponding to inter-planar distance between C_3_N_4_ layers (Figure S5a).^[7]^ The X-ray photoelectron spectroscopy (XPS) C 1*s* spectrum showed a large peak at 288.2 eV, which corresponds to the N=C–N moieties in the triazine building units (Figure S5b).^[8]^ The deconvoluted N 1*s* spectrum of UCN showed peaks at 398.8, 400.1, and 401.4 eV, corresponding to the C=N–C, –N=N–/N–(C)_3_, and –NH_x_, respectively (Figure S5c).^[7]^ The Fourier transform infrared (FT-IR) spectrum of the UCN showed peaks at 1400 – 1650 cm^−1^, corresponding to heptazine-derived repeating units, and at 1321 and 1250 cm^−1^, corresponding to completely condensed C–N and partially condensed C–NH moieties, respectively (Figure S6).^[9]^ All these characterizations indicate the generation of C_3_N_4_ network in the UCN.


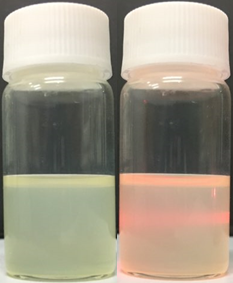
**Figure S1.** Photos for (left) an MD-UCN THF dispersion after one month and (right) its Tyndall effect.

**
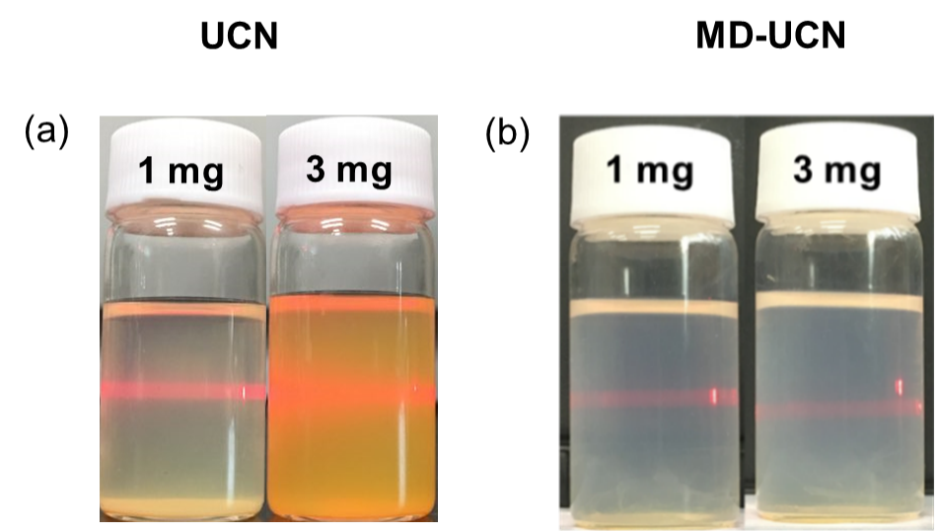
**

**Figure S2.** Photos of suspensions in THF (7 mL) with the Tyndall effect of (a) UCN and (b) MD-UCN with different weight of dispersions.


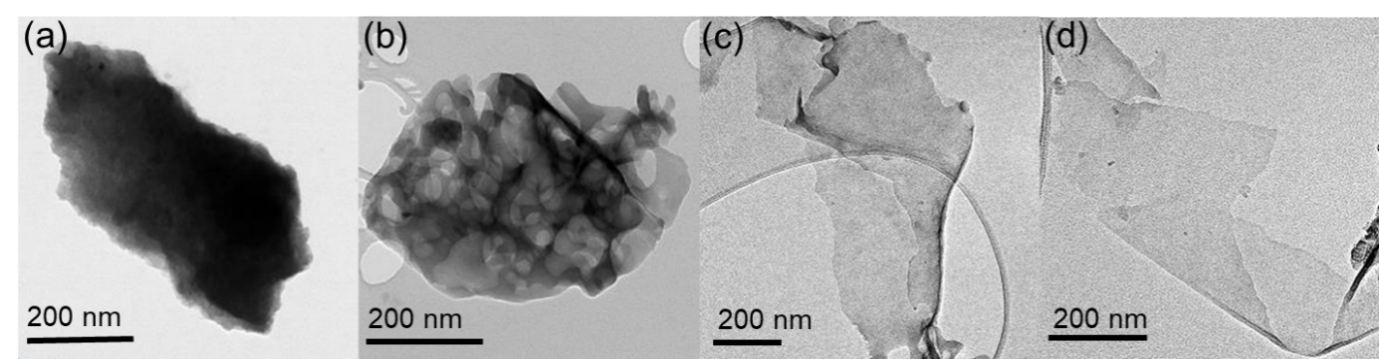


**Figure S3.** TEM images of 3D UCN (a, b) and 2D MD-UCN (c, d) samples, which were prepared with dried droplets of suspensions in THF.


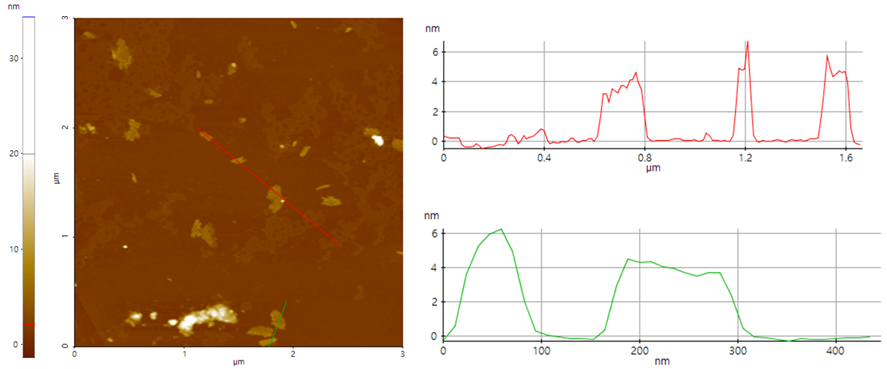


**Figure S4**. An AFM scan of thin 2D MD-UCN materials on a silicon wafer and height profiles.


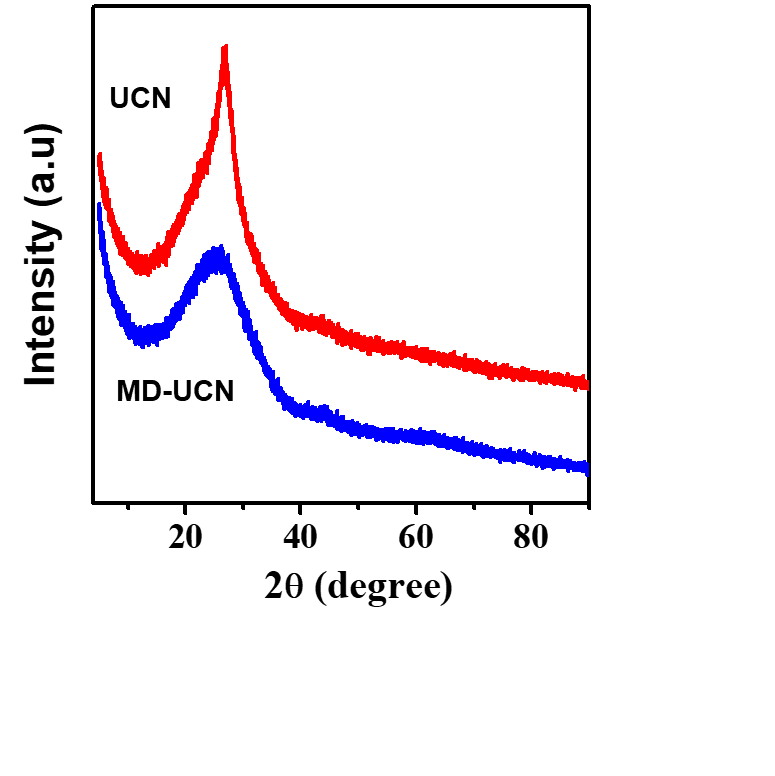
**Figure S5** XRD spectra of 3D UCN and 3D MD-UCN


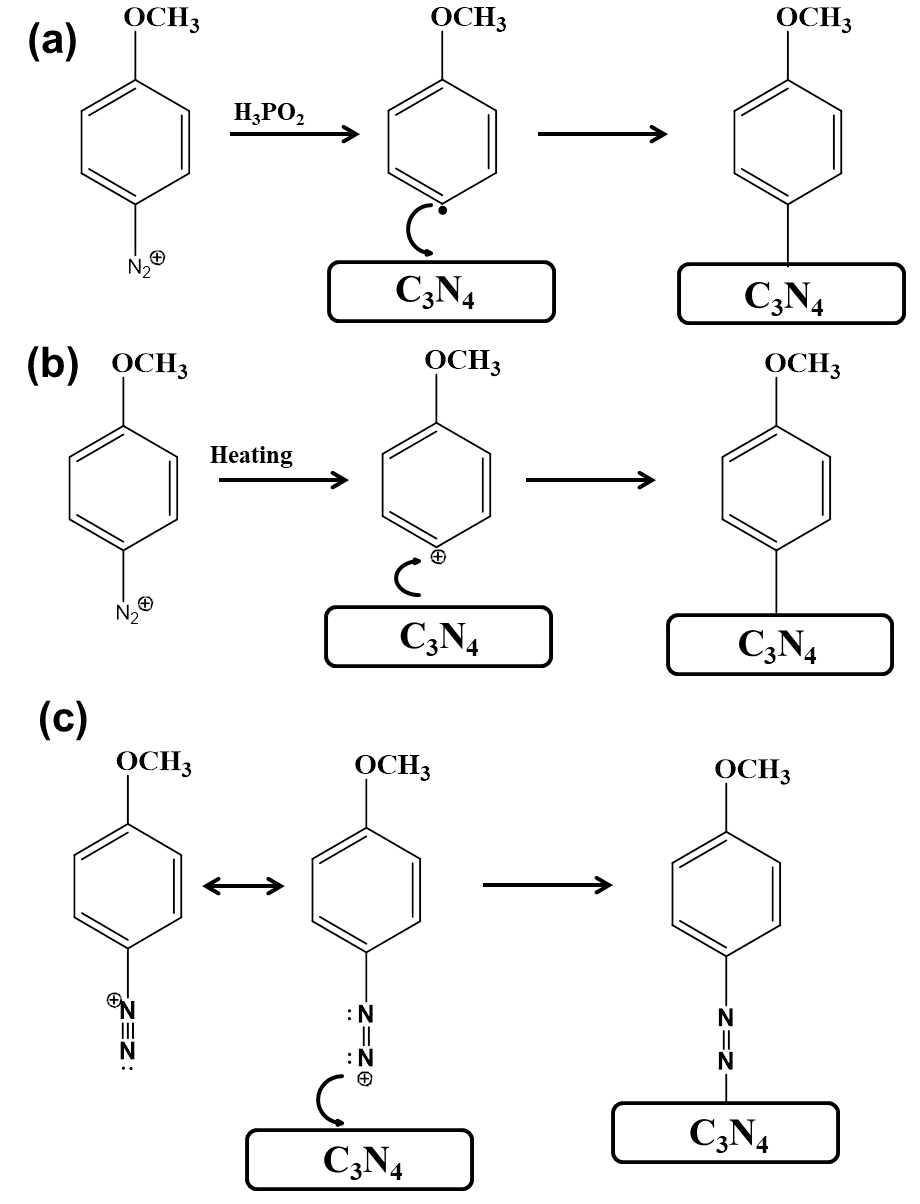
**Figure S6**. Possible mechanisms for the attachment of methoxy-phenyl groups. (a) a C-C coupling reaction through the generation of radicals, (b) a C-C coupling reaction through the generation of carbocations, and (c) an azo-coupling reaction.

Because no H_3_PO_2_ was used in our experiments, the C-C coupling reactions through radical generation did not happen in our reaction (Figure S6a).^[10]^ Two reaction mechanisms for the attachment of the methoxy-phenyl groups are possible in our process;^[11]^ (i) the C-C coupling reaction through carbocations generated by the elimination of –N_2_ groups (Figure S6b) and (ii) the azo-coupling reaction producing a –N=N– bridge between C_3_N_4_ and functional groups (Figure S6c).^[12]^


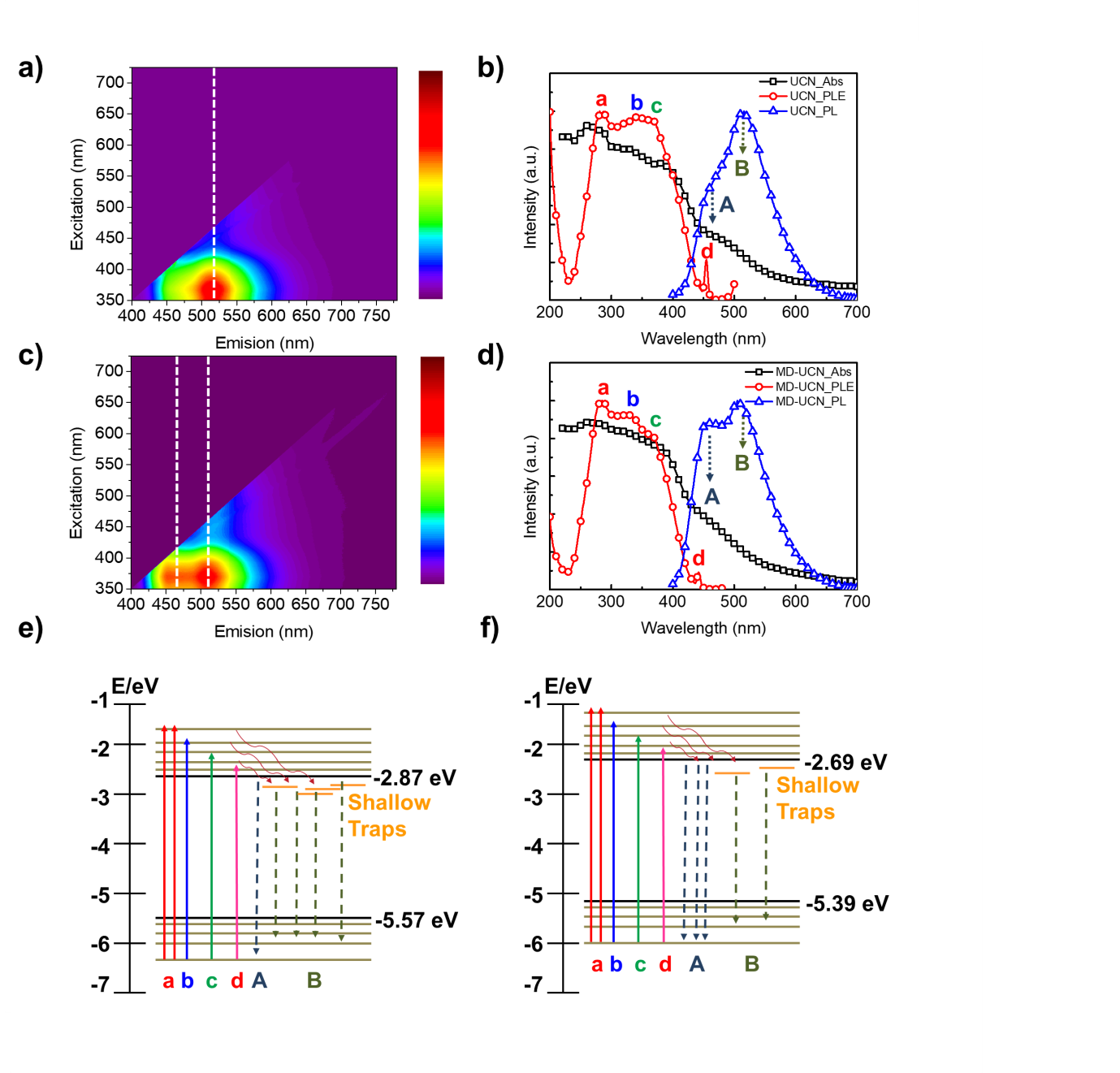


**Figure S7**. (a) PL matrix and (b) absorption (Abs), PL excitation (PLE), and PL spectra of UCN, (c) PL matrix and (d) Abs, PLE and PL spectra of MD-UCN dispersions, and schematic illustrations of the energy structure and emission mechanisms of (e) 2D UCN and (f) 2D MD-UCN.

First, PL spectrum (blue curves) result from recombination of exciton from either band-edge (PL peak at ~460 nm, Blue “A” in the diagram) or defects (PL peak at ~505-517 nm, Green “B” in the diagram). We attribute the largely increased PL peak at 460 nm in MD-UCN to the defect passivation by MD treatments. Second, in PL excitation (PLE) spectrum (red curves), we can assign the electronic transition of electrons from the valence band to the conduction band. Because the energy level of *n* orbitals is substantially influenced by bonding states and surroundings, the C_3_N_4_ has many different energy gaps, each of which interacted with a specific range of excitation light; as a result, the PLE spectrum is broad because it is the sum of many lines. Distinct peaks in PLE (a, b, c, d in the diagram) may be associated with the photoexcitation of electros and thus we draw the schematic illustration of each photoexcitation in the Figure S7e and S7f. Third, the onset of absorption curve (black curves) indicates the optical bandgap of UCN and MD-UCN.


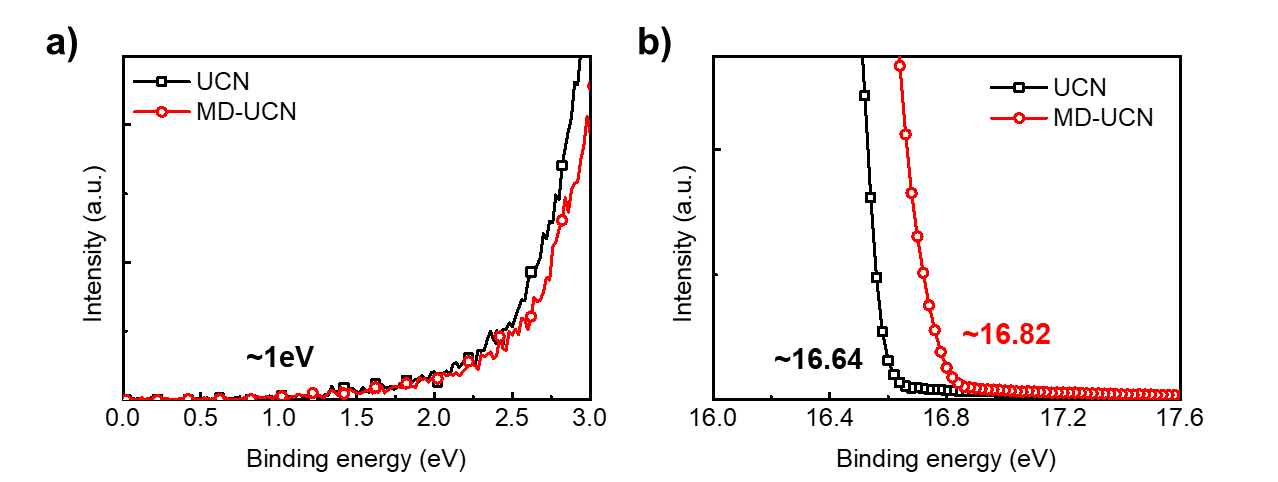


**Figure S8.** (a) Offsets between work function and valence band maximum and (b) secondary cut-off of UCN and MD-UCN measured by UPS (samples were prepared as films on ITO/glass substrates using their dispersions)

The conduction band energy (E_c_) of MD-UCN was calculated to be 2.97 eV by considering valence band energy (E_v_ = 5.39 eV) and energy gap (E_g_ = 2.42 eV) (Figure 5a) calculated from UPS (Figure S9) and UV-Vis absorption spectroscopy (Figure S7b, d), respectively.

**Figure S9**. Photo-stability of MD-UCN and UCN colloidal dispersions measured at their maximum wavelength each under Xe-lamp excitation with wavelength of 370 nm.


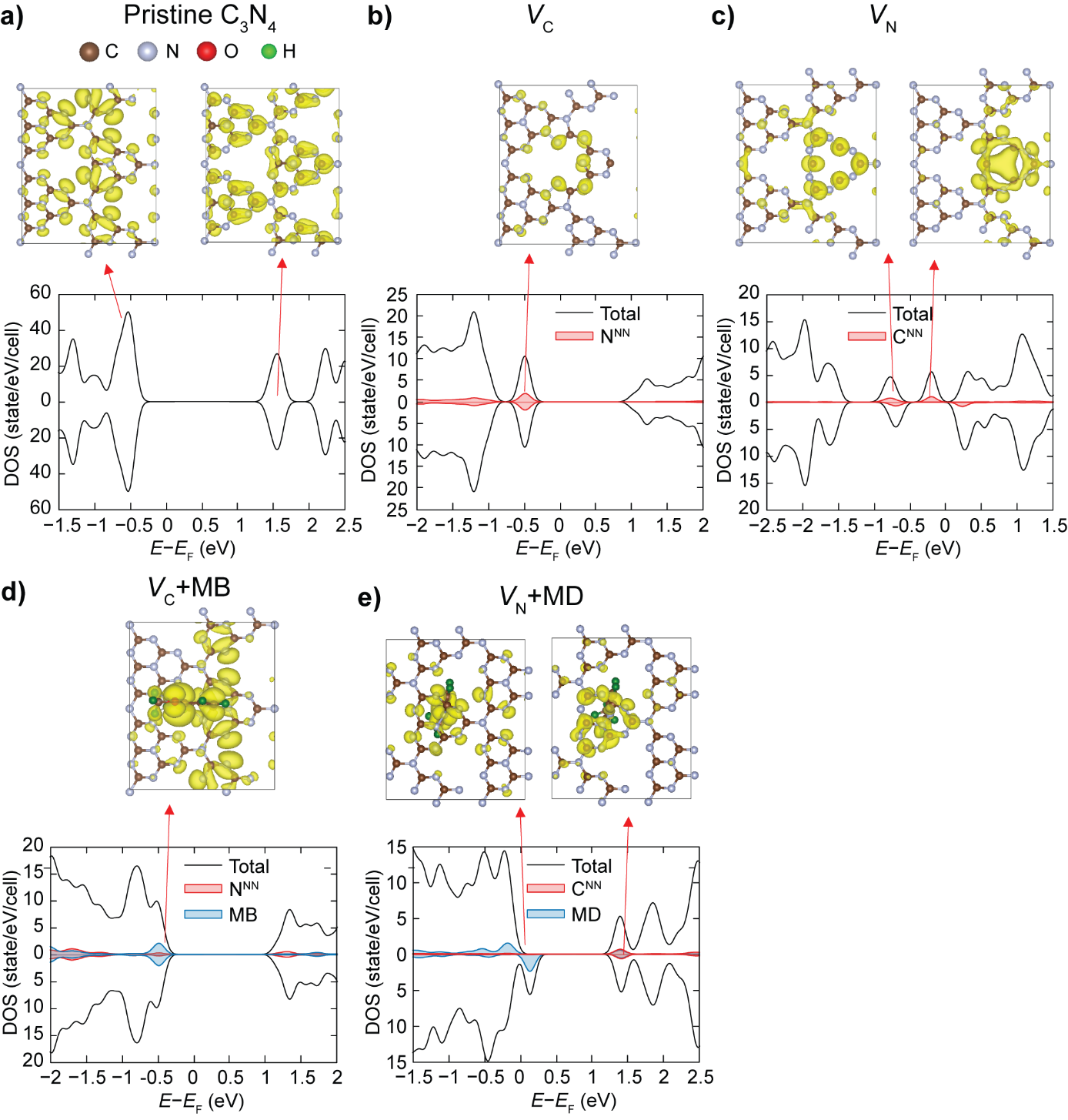


**Figure S10.** The partial charges of important energy levels in (a) pristine C_3_N_4_, (b) *V*_C_, (c) *V*_N_, (d) *V*_C_+MB, and (e) *V*_N_+MD.


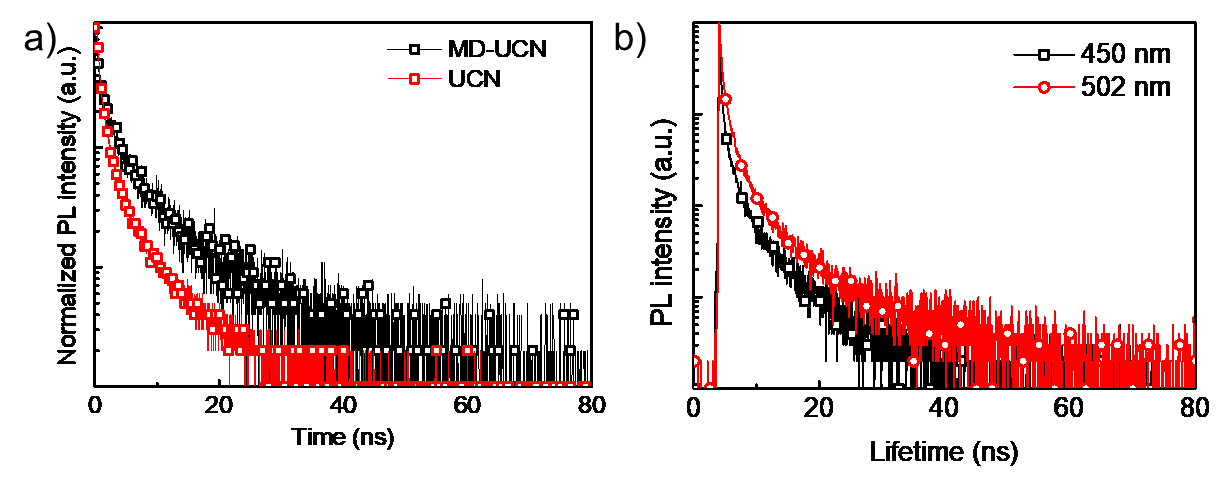


**Figure S11**. Time-resolved PL spectrum of MD-UCN and UCN colloidal dispersions measured at their maximum wavelength each.


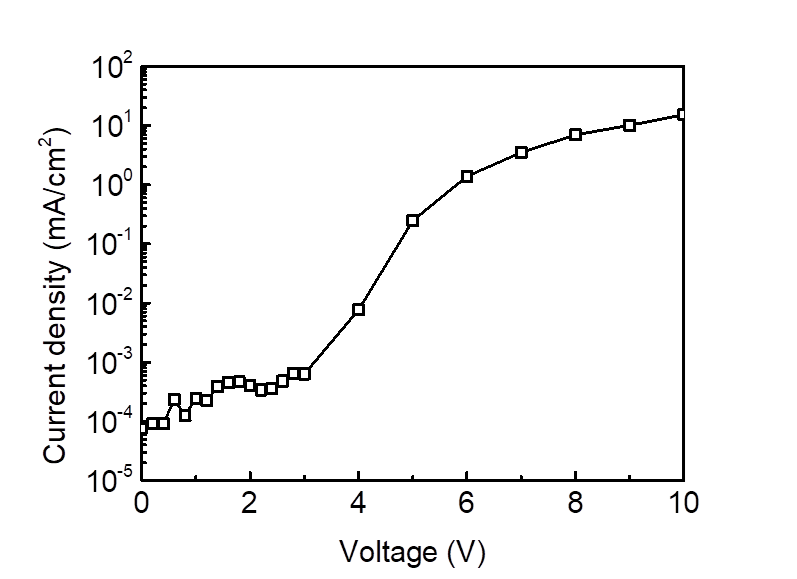


**Figure S12**. Current density-versus-voltage characteristics of MD-UCN LED devices.

**Figure S13**. PL lifetime of 2D MD-UCN dispersions measured at different wavelength.

**Table S1**. Elemental amounts of 3D UCN and 3D MD-UCN samples measured by XPS.

| Sample | C (at%) | N (at%) | O (at%) |
| --- | --- | --- | --- |
| **UCN** | 47.7 | 47.2 | 5.2 |
| **MD-UCN** | 45.6 | 48.7 | 5.8 |

**Table S2**. Relative amounts of O-containing species calculated from the deconvoluted O1*s* XPS spectra of 3D UCN and 3D MD-UCN.

| Sample | C=O | C-O | HO-C=O |
| --- | --- | --- | --- |
| UCN | 55.3 | 35.3 | 9.5 |
| MD-UCN | 48.2 | 43.1 | 8.8 |

**Table S3**. Relative amounts of N-containing species calculated from the deconvoluted N1*s* XPS spectra of 3D UCN and 3D MD-UCN.

| Sample | C=N-C | N-(C)_3_/-N=N- | -NH_x_ |
| --- | --- | --- | --- |
| UCN | 62.7 | 29.4 | 7.9 |
| MD-UCN | 61.1 | 33.2 | 5.6 |

**References**

[1] J. Oh, R. J. Yoo, S. Y. Kim, Y. J. Lee, D. W. Kim, S. Park, *Chem. Eur. J.* **2015***, 21*, 6241.

[2] J. K. Kim, S. Park, R. J. Yoo, H. J. Jeong, J. Oh, Y. J. Lee, S. Park, D. W. Kim, *Chem. Eur. J.* **2018***, 24*, 3506.

[3] P. Allongue, M. Delamar, B. Desbat, O. Fagebaume, R. Hitmi, J. Pinson, J. Saveant, *J. Am. Chem. Soc.* **1997***, 119*, 201.

[4] J. L. Bahr, J. Yang, D. V. Kosynkin, M. J. Bronikowski, R. E. Smalley, J. M. Tour, *J. Am. Chem. Soc.* **2001***, 123*, 6536.

[5] M. S. Strano, C. A. Dyke, M. L. Usrey, P. W. Barone, M. J. Allen, H. Shan, C. Kittrell, R. H. Hauge, J. M. Tour, R. E. Smalley, *Science* **2003***, 301*, 1519.

[6] P. Abiman, G. G. Wildgoose, R. G. Compton, *Int. J. Electrochem. Sci.* **2008***, 3*, 104.

[7] J. Oh, J. M. Lee, Y. Yoo, J. Kim, S. Hwang, S. Park, *Appl. Catal. B: Environ.* **2017***, 218*, 349.

[8] A. P. Dementjev, A. d. Graaf, Sanden, M. C. M. van de, K. I. Maslakov, A. V. Naumkin, A. A. Serov, *Diamond Relat. Mater.* **2000***, 9*, 1904.

[9] H. Zhao, H. Zhao, H. Yu, X. Quan, S. Chen, Y. Zhang, H. Wang, *Appl. Catal. B Environ.* **2014***, 152-153*, 46.

[10] B. D. Assresahegn, T. Brousse, D. Bélanger, *Carbon* **2015***, 92*, 362.

[11] A. Mesnage, X. Lefèvre, P. Jégou, G. Deniau, S. Palacin, *Langmuir* **2012***, 28*, 11767.

[12] G. Schmidt, S. Gallon, S. Esnouf, J. Bourgoin, P. Chenevier, *Chem. Eur. J.* **2009***, 15*, 2101.
